# Bacteriophages are more virulent to bacteria with human cells than they are in bacterial culture; insights from HT-29 cells

**DOI:** 10.1038/s41598-018-23418-y

**Published:** 2018-03-23

**Authors:** Jinyu Shan, Ananthi Ramachandran, Anisha M. Thanki, Fatima B. I. Vukusic, Jakub Barylski, Martha R. J. Clokie

**Affiliations:** 1 Department of Infection, Immunity, and Inflammation, University of Leicester, Leicester, LE1 9HN UK; 2 Department of Molecular Virology, Faculty of Biology, Adam Mickiewicz University, 61-614 Poznan, Poland

## Abstract

Bacteriophage therapeutic development will clearly benefit from understanding the fundamental dynamics of *in vivo* phage-bacteria interactions. Such information can inform animal and human trials, and much can be ascertained from human cell-line work. We have developed a human cell-based system using *Clostridium difficile*, a pernicious hospital pathogen with limited treatment options, and the phage phiCDHS1 that effectively kills this bacterium in liquid culture. The human colon tumorigenic cell line HT-29 was used because it simulates the colon environment where *C. difficile* infection occurs. Studies on the dynamics of phage-bacteria interactions revealed novel facets of phage biology, showing that phage can reduce *C. difficile* numbers more effectively in the presence of HT-29 cells than *in vitro*. Both planktonic and adhered *Clostridial* cell numbers were successfully reduced. We hypothesise and demonstrate that this observation is due to strong phage adsorption to the HT-29 cells, which likely promotes phage-bacteria interactions. The data also showed that the phage phiCDHS1 was not toxic to HT-29 cells, and phage-mediated bacterial lysis did not cause toxin release and cytotoxic effects. The use of human cell lines to understand phage-bacterial dynamics offers valuable insights into phage biology *in vivo*, and can provide informative data for human trials.

## Introduction

The increase in antibiotic resistance found within multiple pathogenic bacteria has motivated an interest in the use of phages to control and treat bacterial pathogens^1–6^. The bacterium *C. difficile* is naturally resistant to many antibiotics and is a leading cause of antibiotic-associated diarrhoea^7,8^. The pathogenesis of the disease is largely caused by two potent *C. difficile* toxins: TcdA and TcdB^9–11^ and the clinically relevant *C. difficile* ribotype R027 produces both toxins at high levels^12^. Three antibiotics are currently used to treat *Clostridium difficile* infection; metronidazole, vancomycin and more recently fidaxomicin^13^. High rates of disease recurrence (20%) are often observed following vancomycin treatment or withdrawal from treatment, and the antibiotics can cause a major disruption to the patients’ microbiota^14^. To further aggravate the situation, *C. difficile* has developed resistance to these antibiotics^15,16^. To avoid a ‘post-antibiotic era’, and to mitigate the complications associated with antibiotic treatments, there is a need for alternative, non-antibiotic approaches to treat bacterial infections and one such approach is phage therapy, the clinical application of phages to treat bacterial infections.

Paramount to phage therapeutic development is evaluating the safety and efficacy of phages that will be used. Currently, this information is mainly obtained from animal models. Successful examples include administration of phages to market-weight pigs contaminated with *Salmonella* Typhimurium, where phages were shown to significantly reduce cecal *Salmonella* concentrations (95%; P < 0.05)^17^. Another study used a mouse model to show that phage treatment significantly decreased the mortality of thermally injured, *Pseudomonas aeruginosa*-infected mice^6^.

With respect to *C. difficile* phage development, progress has been made in understanding the fundamental biology of *C. difficile* phages with regards to their growth dynamics, host specificity, morphologies, and genomes^18–26^. The safety and efficacy of therapeutic phage treatment of *C. difficile* infections have also previously studied using hamster, artificial gut, and more recently the insect *Galleria mellonella* models^2,27–30^. The findings from these studies suggested phage treatment appeared to be safe and effective; hamster data showed that they responded positively to phage treatment, and artificial gut model data showed that *C. difficile* numbers and toxin levels were reduced with minimal disruption to commensal bacteria. Furthermore, a combination of four *C. difficile* phages eliminated *C. difficile* in the insect *Galleria mellonella* model^29^.

Although animal models, such as those discussed above are useful to determine the safety and efficacy of phages, the sole use of animals to predict *in vivo* activity of phages in humans is expensive and time-consuming. For example, it is common for the *in vitro* antimicrobial activity of phages to not be directly translate to *in vivo* phage lysis in animal models^31,32^. Clearly, more efforts are needed to understand the *in vivo* phage activity before moving to animal models and human trials. One way to evaluate phages effectiveness is to use a suitable *in vitro* system, which can provide data on the *in situ* dynamics between phages, bacteria and mammalian cells.

Human cell lines have been used routinely as models to predict clinical responses to drugs, and for drug screening/toxicity studies^33–36^. In addition, a large body of literature on bacteria and human cell lines has shown how bacteria attach and grow in the presence of human cells^37–40^. Of particular pertinence to this work are, several studies that have used cell lines to assess certain aspects of phage therapy. For example, in one study Alemayehu *et al*., (2012) demonstrated that phages effectively killed *Pseudomonas aeruginosa* growing on a cystic fibrosis bronchial epithelial cell line^41^. In another study, a lung epithelial cell was used to measure the safety of phage treatment of *Acinetobacter baumanni*^42^. Recent work by Mirzaei *et al*., (2016) investigated the immune responses to *E. coli* phages using epithelial cell lines HT-29 and Caco-2, revealing different levels of immunogenicity that were seen in response to four distinct phages^43^. These studies revealed the importance of cell-line studies to examine safety aspects of phages and the immune responses. However what distinguishes the work presented here from work that has been previously published is that previous studies have focused on how phages interact with bacteria, OR human cells but we have assessed all three components and attempted to describe the factors that govern the dynamics between bacteria, phages and human cells.

A major strength of human cell lines as a tool to study phages is that *in vivo* data on the mechanisms of interactions between bacteria and phages can be obtained. Although in phage therapy phages would be given as antimicrobials, it is worth remembering that within the human body is vast and diverse microbiome that these ‘added’ phages would interact with. Metagenomic studies have revealed that human intestines have approximately ten times more bacterial cells than human cells^44^, and a further approximately ten times more phages^45^. Phages are naturally found on intestinal mucosal membranes and on dermal surfaces where they are thought to boost the immune system by preventing bacterial colonisation^46,47^. Despite the vast number of phages in the GI tract, there is limited knowledge on their roles in intestinal bacterial pathogenesis. Our lack of knowledge regarding intestinal phages echoes our poor understanding of marine phages in the 1990 s. Detailed studies of marine phages subsequently showed that phages play pivotal roles in marine microbial food webs, structuring bacterial communities, shaping evolution and driving nutrient cycling^48,49^. Studying phages within a human cell culture environment is likely to reveal facets of their biology *in vivo* and explain their significance in a human context. Knowledge gained from experiments with phages, bacteria, and human cells can inform the selection of phages most suited to therapeutic purpose.

In this study, we examined dynamics of the phage phiCDHS1 when used to treat HT-29 cells infected with *C. difficile* ribotype R027 strain. The HT-29 cells were chosen primarily because *C. difficile* colonises the gut and HT-29 was previously used to investigate the pathological effects of *C. difficile*^50–52^. Human cervical cancer cells, HeLa, were also used to test the specificity of phage adsorption to human cells. The phage phiCDHS1 was selected as it was previously shown to effectively kill the widespread epidemic strain of *C. difficile* R027 in bacterial liquid culture^53^.

To summarise, the use of cell cultures to determine the safety and ability of phages to reduce bacterial numbers is inexpensive and can provide useful information about how phages would behave *in vivo*. It clearly provides further information to the current assessment of *in vitro* antimicrobial activity of phages in liquid culture. Human cells are likely to impact the phage-bacteria interactions, yet their impacts on such interactions have been little studied. Indeed no previous publications report data on how human cells impact the ability of phages to adsorb to, and kill bacteria. Our data confirm that human cell lines are indeed valuable tools to study phage *in vivo* activity and can be used to obtain efficacy, toxicity, and mechanistic data. We propose that our model will be a useful tool for phage therapy development and can be adapted to study other bacteria-phage interactions with suitable human cell lines.

## Results and Discussion

### Growth of bacteria, phages, and human cells

A prerequisite to studying phage-bacteria interactions in human cells is optimising the conditions that promote the replication of bacteria, phages and human cells. Moreover, it is vital not to transfer toxins from bacterial cultures into the human cell cultures. To ensure these parameters all held, we confirmed that after washing the bacterial pellets and resuspending them in human cell culture medium, bacteria were viable, free of toxins, and were still susceptible to phage infection.

**Figure 1:**
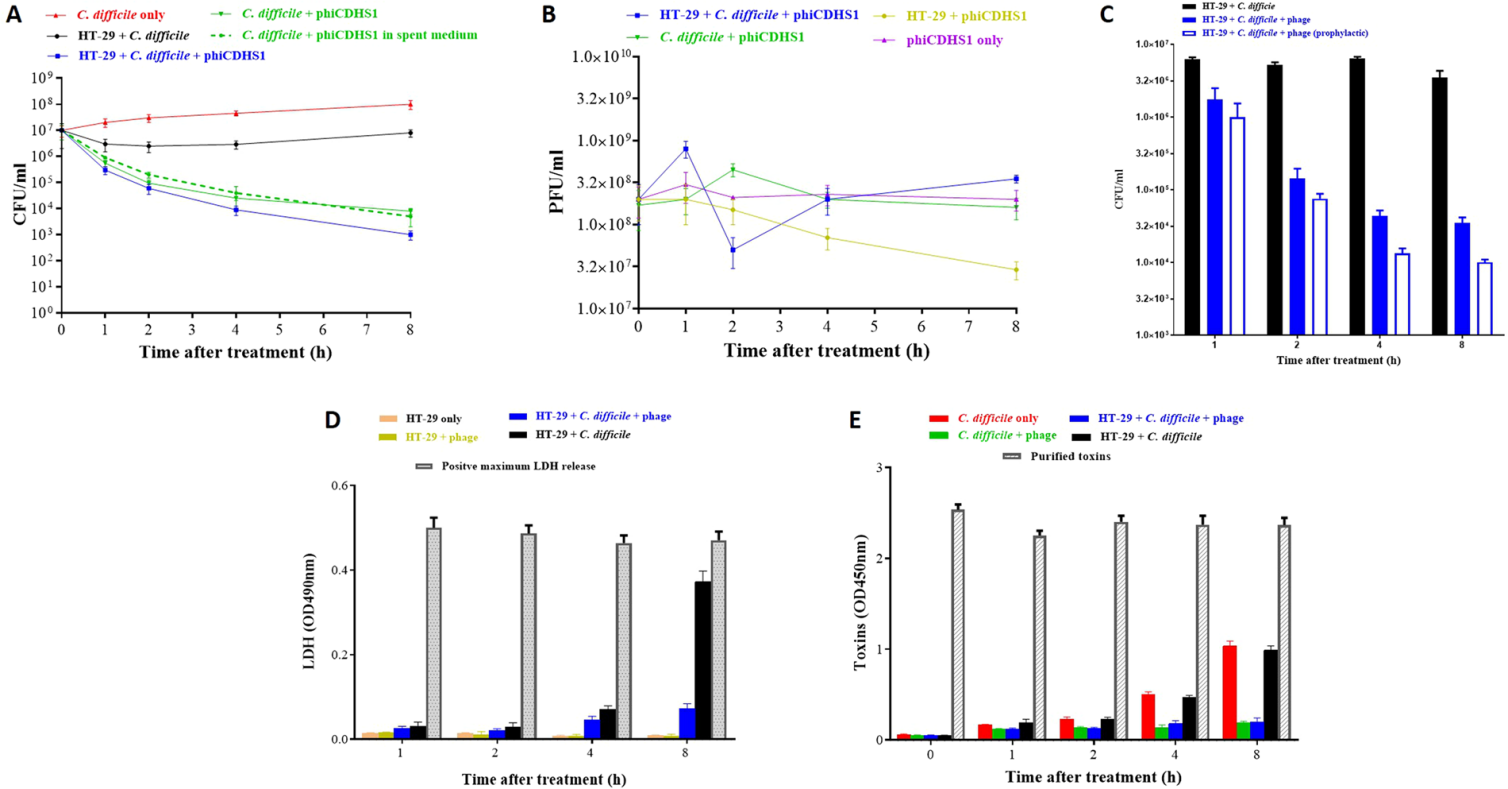
Phage treatment significantly reduced the number of planktonic and adhered *C. difficile* on HT-29 cells and consequently significantly reduced the *C. difficile* toxin production and cytotoxic effect. Monolayers of HT-29 cells (or HT-29 spent medium) in 24-well plates were incubated with *C. difficile* and phage under anaerobic conditions. For prophylactic application, phages were added one hour before bacteria. The number of free/adhered *C. difficile*, free phages, and toxin production were determined at time intervals. To assess the cytotoxic effect of phage treatment of *C. difficile* on human cells, the level of lactate dehydrogenase (LDH) released by HT-29 cells was also determined. (**A**) The concentration of free *C. difficile* (CFU/ml). (**B**) The titre of free phages (PFU/ml). (**C)** The concentration of adhered *C. difficile* (CFU/ml). (**D**) The level of LDH measured as OD_490_. (**E**) The toxin production measured as OD_450_. Mean values of three biological replicates are presented. Error bars denote standard error of the mean.

Typically, human cells are cultured in atmospheric conditions supplemented with 5% CO_2_^37^. However, in the human body, oxygen concentrations naturally range from 0.5 to 7% in the brain; 4–12% in the liver, heart and kidneys; and 3–5% in the uterus, rather than the 21% in the atmosphere^54,55^. Considering the largely anaerobic nature of human intestine, where *C. difficile* resides, setting up an epithelial cell model in anaerobic condition is a close approximation to the conditions found *in vivo*^56^. To overcome the apparent paradox of culturing anaerobic bacteria with aerobic human cells, we confirmed that HT-29 and HeLa cells were able to tolerate anaerobic conditions during an 8 h incubation in anaerobic chamber. The viability of HT-29 and HeLa cells was determined by measuring the level of cytoplasmic lactate dehydrogenase (LDH) released into the medium. As seen in Fig. 1D, a very low level of LDH release (OD_490_ = 0.008 at 8-h, less than 2% of the maximum LDH release) was detected from HT-29 after incubation. An almost identical low level of LDH release was also observed for HeLa cells (data not shown). The LDH value from the aerobic conditions (CO_2_ incubator) was very similar to the anaerobic value (OD_490_ = 0.007 at 8-h). This demonstrates that both HT-29, and HeLa cells can survive anaerobic conditions for experiment duration, therefore the use of the model under anaerobic conditions was deemed suitable for further experiments.

### Dynamics of phage-bacteria interaction with HT-29 cells

To evaluate phage efficacy in the cell culture model, five key parameters were determined and compared to the control without HT-29 cells. These were; bacterial survival, bacterial colonisation, phage production, *C. difficile* toxin production, and HT-29 cell viability. In the absence of HT-29 cells, *C. difficile* increased from approximately 10^7^ to approximately 10^8^ Colony Forming Units (CFU)/ml over the 8-h period (Fig. 1A). In contrast, the phage-infected culture showed a continuous drop in CFU, from approximately 10^7^ to approximately 10^4^ CFU/ml over the same time period (Fig. 1A). The decrease in CFU was due to the phage mediated lysis of *C. difficile*, which was accompanied by a 2-fold increase in free phage titre, Plaque Forming Units (PFU)/ml, at 2-h post infection (Fig. 1B). The PFU peak at 2-h is significantly higher than that in the phage only samples (Fig. 1B, P < 0.05).

In contrast, when the experiment was conducted in the presence of HT-29 cells, a new dynamic was observed. For the non-phage-infected control, the free *C. difficile* number decreased from approximately 1 × 10^7^ to approximately 3 × 10^6^ CFU/ml within 2 h then increased to approximately 7 × 10^6^ CFU/ml at 8 h post infection (Fig. 1A). This initial drop in free CFU was due to the adherence of *C. difficile* to the HT-29 cells. As illustrated in Fig. 1C, approximately 6 × 10^6^ CFU/ml of *C. difficile* was adhered to HT-29 cells throughout the time course. This approximately 60% *C. difficile* adsorbed to HT-29 cells, is comparable to *C. difficile* adsorption to other cell lines^50,57,58^. The increase in the free *C. difficile* number from 2 h onwards therefore is the active growth of free-living planktonic *C. difficile*.

For the samples that contained all three model components (HT-29 cells, *C. difficile* and phages), the number of planktonic *C. difficile* cells decreased more rapidly than in the absence of HT-29 cells, and reached a significantly lower number at 4 h (approximately 10^4^ CFU/ml; P < 0.05) and 8 h (approximately 10^3^ CFU/ml; P < 0.005) post infection (Fig. 1A). Furthermore, the number of adhered *C. difficile* in phage-infected HT-29 sample was also significantly less than that in the non-phage-infected control for all time points (P < 0.05) (Fig. 1C). For example, only approximately 4 × 10^4^ CFU/ml of *C. difficile* attached to HT-29 in phage-infected samples compared to approximately 5 × 10^6^ CFU/ml in non-phage control at 8 h (Fig. 1C). In addition to a CFU reduction, phage production was increased in the three-component system compared to HT-29 and phiCDHS1 only (Fig. 1B, p < 0.001 at 8-h post infection), indicating successful phage production. In two-component system with no phage propagation, the titre of free phages dropped throughout the time course due to their strong adsorption to HT-29 (Fig. 1B). Finally, it was important to highlight that at 8-h post infection, the titre of free phages in the three-component system was significantly higher than the samples comprising *C. difficile* and phiCDHS1(p < 0.05), suggesting human cells promote phage propagation.

Overall, phages in the presence of HT-29 cells seem to be more active at lysing *C. difficile* than phages in the absence of HT-29 cells. To determine if HT-29 secretions benefitted phage efficacy, the killing efficiency of phiCDHS1 against *C. difficile* was determined in fresh and spent HT-29 cell culture media and no differences were observed suggesting that HT-29 cells do not promote phage lysis (Fig. 1A). The marked increased phage activity with HT-29 cells is however likely to be due to the monolayer of HT-29 cells bringing phage and *C. difficile* into close contact, and facilitating their interaction and subsequent phage action.

### Phage adsorption to human cells

It is known that *C. difficile* has a strong tendency to adhere to human cells^57,59–61^. Several previous reports have also indicated the potential interactions between phages and eukaryotic cells^46,62–64^. However, no attempt was previously made to estimate the phage adsorption to human cells, the first step within phage-human cell interactions, which may hold the key to understanding the behaviour of phages inside human bodies. To establish the proportion of phages that were bound to the HT-29 cells, the number of free phages was assayed using standard methods to calculate phage adsorption to bacteria^65–68^. In the samples comprising phage and HT-29 cells, the free phages were measured to be approximately 3 × 10^7^ PFU/ml at 8 h compared to the total input of 10^8^ PFU/ml (P < 0.05) (Fig. 1B). Therefore, approximately 70% of total phages (7 × 10^7^/10^8^ × 100) were bound to HT-29 cells.

**Figure 2:**
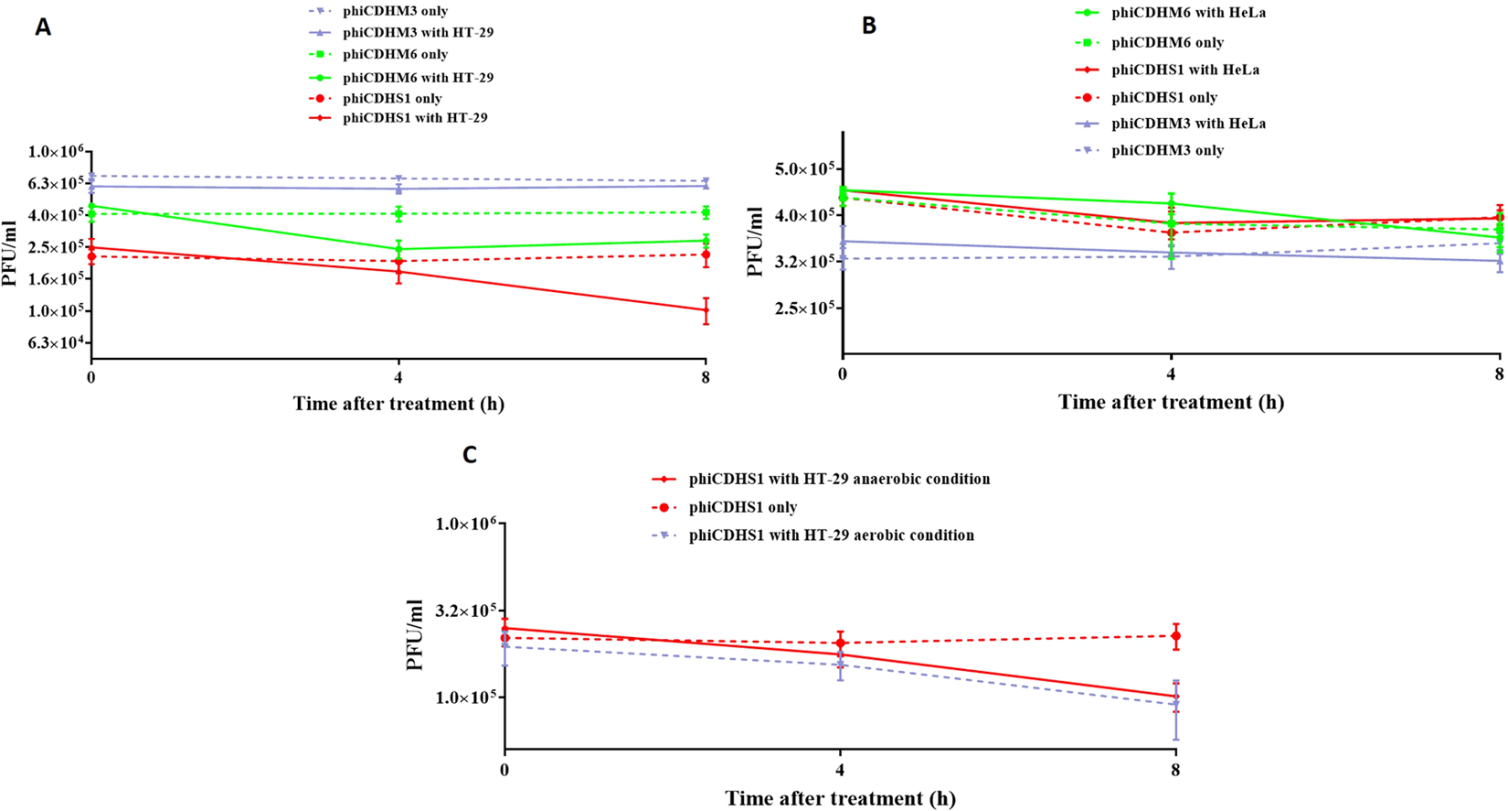
Adsorption of phiCDHM3, phiCDHM6, and phiCDHS1 to HT-29 and HeLa cells, respectively. Phages were added onto monolayers of HT-29/HeLa cells in 24-well plates. The titres of free phages were determined at time intervals (PFU/ml). (**A**) phiCDHS1 and phiCDHM6 showed approximately 70%, and 40% adsorption to HT-29, respectively. While phiCDHM3 showed no noticeable drop in the number of free phages, which demonstrated no adsorption to HT-29. (**B**) The titres of free phages determined at time intervals remained unchanged. This established that all three phages were unable to adsorb to HeLa cells. (**C**) phiCDHS1 adsorption to HT-29 cells under both anaerobic and aerobic conditions. Mean values of three biological replicates are presented. Error bars denote standard error of the mean.

To investigate if this marked phiCDHS1 adsorption to HT-29 cells is general or specific, three different *C. difficile* phages (phiCDHS1, phiCDHM3 and phiCDHM6) were tested against two cell lines, HT-29 and HeLa cells. Compared to 70% adsorption of phiCDHSI to HT-29, 40% of phiCDHM6 bound to HT-29, while phiCDHM3 showed no adsorption to HT-29 (Fig. 2A). Furthermore, none of the three phages were able to bind to HeLa cells (Fig. 2B), which could reflect the specificity of phage adsorption to human cells as *C. difficile* phages are more likely to be naturally found in the gut than uterus. The specific phage adherence to human cells is somewhat analogous to phage adsorption to bacteria, which is governed by the specific interaction between the phage and the receptor distributed over the bacterial surface. Therefore, it is logical to hypothesise that phage adsorption to human cells may depend on specific interactions between phages and receptors located on human cells. To date no ‘phage receptors’ have been reported or identified in mammalian cell surfaces. One possible mechanism that has been suggested is that the major capsid protein of *E. coli* phage T4 may interact with β3 integrins of target cells^69^. Further research is needed to determine the nature of the human cell receptors for phages. It is also worth mentioning that there were no significant difference regarding the adsorption dynamics of phiCDHS1 to HT-29 in anaerobic or aerobic conditions (Fig. 2C).

The ability of phages to colonise the epithelial cells suggests that prophylactic phage application could lead to good protection from bacterial infection. Thus, we predicted that fewer adhered *C. difficile* would be observed if HT-29 cells were pre-incubated with phages. Indeed, when phages were added to HT-29 cells one hour before the addition of *C. difficile*, the number of colonised *C. difficile* was approximately 10^3^ CFU/ml at 8 h post infection compared to approximately 4 × 10^4^ CFU/ml when phage and *C. difficile* were added at the same time (Fig. 1C, P < 0.05).

The ability of phages to adsorb to human cells and to prevent *C. difficile* colonisation has important implications in phage therapy in general, and suggests that applying phage prophylactically would achieve better protection than exploiting phages as a therapeutic treatment. A recent *C. difficile* phage therapy study conducted in *Galleria mellonella* indeed showed that the prophylactic groups receiving phages before *C. difficile* inoculation displayed higher survival rate than phage treatment groups^70^.

### Cytotoxic response of HT-29 cells

A further advantage of using epithelial cell lines to assess phage therapy is that the efficacy and safety of phage treatment can be measured in the same experiment. As seen in Fig. 1D, phages showed no cytotoxic effect on HT-29 cells as an almost identical LDH readings were observed in the sample containing phage and HT-29 cells and the sample containing HT-29 only. With respect to the groups consisting of *C. difficile*, HT-29 and phages, the level of LDH was consistently lower than that of the *C. difficile*-infected HT-29 groups with a significantly lower LDH release was observed at 8-h post phage treatment (Fig. 1D, p ≤ 0.0001). The results demonstrate that phage treatment has significantly reduced the cytotoxic effect of *C. difficile* on HT-29 cells.

The final data presented in this paper is consistent with phages removing bacteria in a ‘non-harmful’ way, i.e, phage-mediated bacterial lysis doesn’t lead to undesirable side effects such as toxin release. Significantly low levels of *C. difficile* toxins were observed with phage-treated samples compared to the non-phage-treated samples (P < 0.0001). The lack of toxin production, which may be caused by lysing exponentially growing vegetative *C. difficile* cells before they can reach the toxin-producing stationary phase of bacterial growth^71^, explains the reduced cytotoxic effect in phage treated samples.

### Use of human cells in phage research

Human cell lines have been used extensively in high-throughput screening of drugs before animal experimentation is carried out^33–36^. Despite their wide use in this context, currently, only a very few studies used human cell lines to determine the safety and immune responses of phages^41–43^. Furthermore, to the best of our knowledge no previous studies have been published that use all the advantages of *in vitro* cell lines as inexpensive and convenient models to reveal the interactions of phages, bacteria and human cells. In this study, we established a methodology to investigate the suitability of phages to treat *C. difficile* infection in human epithelial cells. In comparison to standard broth-based *in vitro* phage killing assays, the cell culture-based assays revealed novel dynamics between phage-bacteria. For example, phages are more effective against *C. difficile* in HT-29 cells owing to a strong phage binding to the human cells. Further phage binding experiments revealed that different phages had different adsorption dynamics to the same human cell line, and the same phage had different adsorption patterns to different human cells. The specific recognition between phages and phage receptors on human cells are hypothesised to explain the distinctive patterns of phages binding to human cells.

Although the cell culture model cannot provide a direct measure of phage *in vivo* kinetics or pharmacokinetics, it will be able to be exploited as a valuable tool to establish information on the dynamics at the infection site. The subject of phage pharmacokinetics is very poorly understood and again use of human cell cultures in assessing phage-bacteria interaction will provide information to inform *in vivo* work.

In summary, we have shown that human cell cultures can be used as models to assess the safety and efficacy of phage treatment. It is practical and logical to determine phage activity within a representative complex environment (human cells) that simulates the interactions between bacteria and human cells. The methodology developed in the current study can be adapted and applied to other human cell lines and pathogens. We are aware of the limitations of the current model system, such as the relatively short time course, the anaerobic condition, and lack of mucus layers. We are currently working toward further developing this cell culture-based model to reflect the *in vivo* conditions, such as using mucus-producing HT-29 cells and 3D cell cultures. Considering the extensive variety of established human cell lines, and phage-bacteria models there is a significant potential to use human cells to mimic *in vivo* conditions to test phage therapy for many pressing bacterial infections that require novel interventions.

## Methods

### Culture and strain information for *C. difficile*, phage phiCDHS1 and human epithelial cell line HT-29 and human cervical cancer cell line HeLa

The *C. difficile* strain CD105LC1 was isolated from patient faecal samples obtained from the University Hospitals of Leicester NHS Trust, and shown to be a PCR ribotype 027 (R027)^24^. The bacterium was grown in Brain Heart Infusion (BHI) medium under anaerobic condition at 37 °C in a Don Whitely Anaerobic Chamber (UK).

The phage phiCDHS1 was previously isolated from estuarine sediments using the CD105LC1 strain^72^. Phages phiCDHM3 and phiCDHM6 were characterised before by Nale, *et al*., 2017, and were prepared according to the previous published method^28^. Phage titration was performed using the soft agar overlay method supplemented with MgCl_2_ and CaCl_2_ salts^25^.

Human colon carcinoma HT-29 and human cervical cancer cells HeLa were obtained from European Collection of Cell Cultures (ECACC) and were grown in cell culture medium comprising of Dulbecco’s modified Eagle’s medium (DMEM) supplemented with 2% foetal bovine serum (FBS) (v/v) and 2 mM L-glutamine. Cells were cultured at 37 °C and 5% CO_2_ in a humidified environment. The spent medium was referred to as the cell-free supernatants from HT-29 cell cultures with 90% confluence.

### Measurement of planktonic and adhered bacteria and free phages

Both planktonic bacteria and free phages were measured according to previously established methods^72^. Briefly, to measure the number of bacteria attached to the HT-29 monolayer, the wells were washed three times with 1 mL of PBS to remove non-adherent bacteria. The monolayers were then treated by adding 1 ml of PBS containing 0.1 mM EDTA for 10 mins at 37 °C followed by vigorous pipetting. The number of bacteria present in the homogenous suspensions were then counted and expressed as CFU/ml.

### Measurement of *C. difficile* toxins

As *C. difficile* toxins are primarily responsible for cytotoxic effect, the toxin levels in phage-infected and non-infected samples were determined^15,17^. C. DIFFICILE TOX A/B II™ (TECHLAB) was used for measuring toxin A and toxin B. The manufacturer’s instructions were followed, and the toxin levels are expressed as OD_450_ with positive and negative controls provided by the kit.

### Cell cytotoxicity test

The safety and the impact of phage treatment on the viability of HT-29 cells were measured using the CytoTox 96 assay kit (Promega). This assay measures the levels of cytoplasmic lactate dehydrogenase (LDH) released into the cell culture medium, which is positively correlated to the cell membrane damage. The more cells that are lysed, the more LDH is released. The amount of LDH was visualised through LDH enzyme activity of converting a tetrazolium salt into a red formazan product. The amount of red colour therefore is proportional to the number of lysed HT-29 cells. The value of optical density at 490 nm was then used to indicate the level of cytotoxicity^73,74^. The manufacturer’s instructions were followed with modifications. Briefly, 50 µl of supernatants were transferred into 96-well plates followed by 50 μl of the assay buffer containing the detection dye and the catalyst. After 30 min of incubation at room temperature, 50 μl stop solution was added and the absorbance at 490 nm was measured.

### Optimisation of parameters and experimental setup

This was achieved by washing metabolically active *C. difficile* cells in cell culture medium to remove toxins^57,75^. Specifically, exponentially growing cultures of *C. difficile* (OD_550_ of 0.3) were washed twice in cell culture medium by centrifugation at 4,500 × *g* for 15 min at room temperature and then re-suspended in this medium. Phage lysates were washed twice in cell culture medium by centrifugation at 21,000 × *g* for 30 min at 4 °C. The final phage pellet was re-suspended in 1 ml of cell culture medium.

The aerotolerance of human cells has been reported previously, for example human mesenchymal stem cells were shown to tolerate anaerobic condition for up to 24 h and retain 80% viability^76^. To determine the ability of HT-29 and HeLa cells to tolerate anaerobic condition, both cells were inoculated in a 24-well plate and incubated under anaerobic and aerobic conditions followed by the LDH-based cell cytotoxicity test.

The starting culture of HT-29 and HeLa was adjusted to 7 × 10^4^ cells/ml using cell culture medium and 1 ml was seeded in 24-well plates. Plates were incubated for two days (and reached approximately 90% confluence). The total number of cells per well was then approximately 1 × 10^6^. The HT-29 plates were used in the following test with *C. difficile* and phiCDHS1. Next, 0.1 ml of *C. difficile* (10^8^ CFU/ml) and/or 0.1 ml of phiCDHS1 suspension (10^9^ PFU/ml) were added to each well of HT-29, including wells in the control plates that had no HT-29 cells; instead 1 ml of cell culture medium was added. For prophylactic phage administration, phages were added 1 h before the bacteria addition. Both experimental and control plates were incubated in an anaerobic chamber. 500 µl of samples (supernatants) were collected immediately at time zero (0-h) and then at 1, 2, 4 and 8-h after infection. These supernatants were used for counting planktonic bacteria (CFU), free phages (PFU), *C. difficile* toxin production and the cytotoxic response of HT-29 cells. Care was taken to not touch the HT-29 monolayer while the supernatants were taken because the monolayer needed to be lysed in parallel to count the adhered bacteria.

For testing phage adsorption to human cells, both HT-29 and HeLa plates were used. Phage adsorption tests were carried out the same way as measuring the dynamics of phiCDHS1, *C. difficile*, and HT-29 except without the involvement of bacteria. Briefly, 0.1 ml of phage suspensions of phiCDHM3, phiCDHM6, and phiCDHS1 were added into each well of HT-29 and HeLa, respectively. Supernatants were collected immediately at time zero (0-h) and then at 4 and 8-h after infection. These supernatants were used for counting phages (PFU/ml).

### Statistical Analysis

The data presented are the means with standard errors from three biological replicates. The statistical significance was assessed using multiple t-tests within Prism GraphPad 4 software. Significance was accepted at P ≤ 0.05.
